# Effect of summer acupoint application treatment (SAAT) on gut microbiota in healthy Asian adults: A randomized controlled trial

**DOI:** 10.1097/MD.0000000000032951

**Published:** 2023-03-03

**Authors:** Jie Zhou, Bangmin Zhou, Xiaoyue Kou, Tao Jian, Limei Chen, Xinghua Lei, Shijian Jia, Xiaoying Xie, Xianbo Wu

**Affiliations:** a Department of Project Management Division, XinDu Hospital of Traditional Chinese Medicine, Chengdu, Sichuan, PR China; b Department of Preventive Treatment, XinDu Hospital of Traditional Chinese Medicine, Chengdu, Sichuan, PR China; c Department of Hepatobiliary Surgery, Jintang First People’s Hospital, Chengdu, Sichuan, PR China; d Department of Acupuncture Rehabilitation, XinDu Hospital of Traditional Chinese Medicine, Chengdu, Sichuan, PR China; e Department of Traditional Chinese Medicine, College of Sports Medicine and Health, Chengdu Sport University, Chengdu, Sichuan, PR China.

**Keywords:** 16S rDNA, gut microbiota, healthy adults, summer acupoint application treatment

## Abstract

Acupoint application has served as an important complementary and adjunctive therapy in China. The purpose of this study is to explore the impact of summer acupoint application treatment (SAAT) on the abundance and biological structure of gut microbiota in healthy Asian adults. Based on the CONSORT guidelines, 72 healthy adults were included in this study, randomly divided into 2 groups, receiving either traditional (acupoint application within known relevant meridians, Group A) or sham (treated with placebo prepared by mixing the equal amount of starch and water, Group B) SAAT. SAAT stickers include extracts from Rhizoma Corydalis, Sinapis alba, Euphorbia kansui, Asari Herba, and the treatment group received 3 sessions of SAAT for 24 months, administered to BL13 (Feishu), BL17 (Geshu), BL20 (Pishu), and BL23 (Shenshu) acupoints. Fecal microbial analyses via ribosomal ribonucleic acid (rRNA) sequencing were performed on donor stool samples before and after 2 years of SAAT or placebo treatment to analyze the abundances, diversity, and structure of gut microbiota. No significant baseline differences were present between groups. At the phylum level, the baseline relative abundance of *Firmicutes, Bacteroidetes, Proteobacteria, Actinobacteria*, and *Fusobacteria* was identified in fecal samples collected from each group. After treatment, the relative abundance of *Firmicutes* was significantly increased in both groups (*P* < .05). Notably, a significant decrease in the relative abundance of *Fusobacteria* was observed in the SAAT treatment group (*P* < .001), while the abundance of *Bacteroidetes* was decreased significantly in the placebo group (*P* < .05). At the genus level, the relative abundance of *Faecalibacterium* and *Subdoligranulum* species in the 2 groups were all significantly increased (*P* < .05). In addition, a significant reduction in the relative abundance of *Blautia, Bacteroides*, and *Dorea* in Group A (*P* < .05) and *Eubacterium hallii* group and *Anaerostipes (P* < .05) in Group B was observed after treatment. Our findings indicated SAAT substantially influenced the bacterial community structure in the gut microbiota of healthy Asian adults, which might serve as potential therapeutic targets for related diseases, and provided a foundation for future studies aimed at elucidating the microbial mechanisms underlying SAAT for the treatment of various conditions such as obesity, insulin resistance, irritable bowel syndrome.

## 1. Introduction

Acupoint application has served as an important complementary and adjunctive therapy in East Asia, and gains wide attention worldwide recently, which has been widely applied in 183 countries according to a 2013 survey.^[1]^ Traditionally, acupuncture contains the rich and profound scientific connotation of Chinese medicine theories that are compatible with Confucianism and Taoism.^[2]^ Currently, there have been various forms of acupuncture applied in several different scenarios, which included electroacupuncture, laser acupuncture, acupressure, auricular needle, knife needle, and moxibustion, etc.^[3]^ Among these therapies, acupoint application treatment (AAT), a modality of traditional acupuncture, as the promising noninvasive procedure combining points and meridians with Chinese herbal medicine has been further refined and generalized in recent years.^[4]^ An experimental study suggested that point application with Angong Niuhuang stickers could improve cognitive function in cerebral ischemic rat models with equivalent efficacy to conventional invasive acupuncture, which is expected to become an economic method for ischemic stroke.^[4]^ Another research indicated AAT with Angong Niuhuang stickers could promote the expression of Bcl-2 and suppress the expression of pro-apoptotic proteins, including Bax and p53 in the hippocampal CA1 area of the cerebral ischemic rat models.^[5]^ Until recently, however, prospective randomized controlled studies regarding the utility of AAT in healthy adults for chronic disease prevention are lacking.

At present, the AAT approach as a route of administration is commonly applied in Asian countries, specifically China and Korea.^[2]^ In Korea, the AAT treatment is often practiced by applying capsicum plaster or other single ingredients on the acupuncture point.^[6–9]^ In contrast, the AAT approach in China is performed by plastering Chinese medicine compounds on an acupoint.^[10–12]^ However, it is noteworthy that the choice of herbal formula and points depends on the heterogeneous disease and syndrome.^[13]^ Indeed, the AAT approach has been applied to treat severe chronic and allergic diseases.^[14–16]^ For example, the basic drugs in the prescription for AAT in summer to treat the pulmonary diseases attacking in winter include Rhizoma Corydalis, Semen Sinapis Albae, Radix Euphorbiae Kansui, and Herba Asari.^[13,17]^ Semen Sinapis Albae is pungent and hot in nature and has the effects of warming the lung, resolving phlegm, eliminating swelling, unblocking the ligaments, and relieving pain, while Herba Asari, is pungent in taste and warm in nature, which could warm the lung, dissolve phlegm, dispel wind, and disperse cold.^[18,19]^ Therefore, AAT with this formula may contribute to the prevention of inflammatory and metabolic diseases.^[20]^ Additionally, Cortex Cinnamomi and Flos Caryophylli are often applied to alleviate painful chronic diseases.^[21,22]^ Correspondingly, the main acupuncture points used in combination include the Bladder meridian (BL), Ren meridian, and Du meridian according to clinical syndrome differentiation.^[13,15]^ Specifically, BL13 (Feishu) is the most fundamental point for AAT, followed by BL17 (Geshu), BL20 (Pishu), and BL23 (Shenshu) acupoints.^[19,23,24]^ In the Chinese medical system, a season-based AAT approach has also been applied to improve or prevent recurrent seasonal diseases.^[25]^ The summer acupoint application treatment (SAAT, also known as “Sanfujiu”) is a way in which herbal compounds are applied in summer at special points (generally in dog days) to prevent active periods or modify diseases attacking in winter, including bronchial asthma,^[11]^ allergic rhinitis,^[14,26]^ and chronic obstructive pulmonary disease,^[16]^ and so forth,^[12]^ and SAAT has been widely used in many provinces in China since the 1950s.^[13]^ A 2-year follow-up study revealed that SAAT on the BL13 and BL12 (Fengmen) acupoints could reduce the frequency and severity of asthma ^[27]^ and SSAT is efficacious in treating seasonal Allergic rhinitis and the treatment efficiency was positively correlated with the length of treatment course.^[25]^ In the realm of traditional Chinese medicine (TCM) theory, SAAT was deemed to be applicable to healthy adults for the purpose of prevention. However, there is limited research focused on the mechanisms of SAAT in the prevention of seasonal diseases.

The gut microorganisms play crucial roles in human health and the imbalance of the gut microbiome is involved in the development of disease. The development of culture-independent, high-throughput sequencing technology of microbial metagenomes has enabled the identification of previously unknown members of the microbiota, thereby providing a powerful new perspective for the composition and function of fecal microorganisms.^[28]^ Recently, studies have found that the occurrence of diseases might be connected to the effects of gut dysbiosis, such as inflammatory bowel disease, rheumatoid arthritis, type 2 diabetes, and obesity.^[29]^ Therefore, gut microorganisms as the potentially modifiable causative factor in the initiation and development of diseases have gained much attention.^[30]^ Considering the importance of gut microbiota, and the pivotal role of gut microbiota in the therapeutic effects of TCM, an increasing number of researches have focused on the interactions between TCM and gut microbiota.^[31]^ Until now, few studies have investigated the effects of SAAT on redressing the disturbance of intestinal microbiota.^[32]^ For these reasons, we designed a randomized controlled study to explore the potential biological effects of SAAT compared to placebo on the composition of fecal microbiota in healthy adults as assessed by 16S ribosomal deoxyribonucleic acid (16S rDNA) sequencing.

## 2. Material and methods

### 2.1. Study design

The aim of this study was to evaluate the efficacy of SAAT on the abundance and biological structure of gut microbiota in healthy Asian adults. This is a randomized controlled trial that participants will be randomly divided into 2 groups (A receiving the SAAT with herbal compound and B receiving placebo). The protocol was approved by the Institutional Review Board of XinDu Hospital of Traditional Chinese Medicine and Chengdu Hanhe Traditional Chinese Medicine Hospital for Preventive Treatment (Approval No. 201801). All participants provided verbal and written consent, and this study conformed to the standards set by the latest revision of the Declaration of Helsinki. The study adheres to the CONSORT guidelines for reporting randomized trials. After participants were enrolled in the study, they were assigned either to study group A or group B on the basis of random numbers generated by an independent investigator. The acupuncturist would give the participant the corresponding intervention according to the random number. All acupuncturists in the study have Chinese medicine practitioner licenses with at least 5 years of clinical experience. The subjects received 3 treatment sessions per year on the hottest days in summer (“Sanfu” Days), according to the lunar calendar, for 24 consecutive months, which was followed by a follow-up period of 6 months. After enrollment, no additional drugs or interventions were allowed during the treatment period. The obligatory acupoints included BL13 (Feishu), BL17 (Geshu), BL20 (Pishu), and BL23 (Shenshu). Fecal samples were collected twice before and after the treatment in both groups and then frozen at −80°C within half an hour after sampling. Specifically, the first sample was collected in the morning before administering the first SAAT or placebo and the second sample was collected in the morning 6 months after the end of treatments. The specimens underwent DNA extraction and 16S rDNA sequencing, which are described below.

### 2.2. Participants

From February 1, 2018, to November 30, 2019, a total of 72 healthy adults were recruited after receiving systematic examinations and randomly distributed into Group A (n = 34) and Group B (n = 38). The inclusion criteria for subjects recruitment included several factors: age 18 to 65 years; no history of acupuncture therapy; medical screening finding good physical health and no meaningful laboratory abnormalities; and providing written informed assent and cooperating with the preventive treatment. The exclusion criteria were as follows: pregnant or lactating women, and those desirous of conceiving in the near future; subjects with organic diseases; subjects with mental disorders; subjects who underwent recent acupuncture or surgical treatments; subjects with skin diseases or defects at the sites of acupoint that prevents the application of SAAT; subjects participating in other clinical trials; and subjects receiving antibiotics, probiotics, prebiotics, TCM, or other drugs within 3 months.

### 2.3. Study intervention and control

For Group A, the subjects were treated with SAAT at each center. The herbal pastes comprised of Rhizoma Corydalis, Semen Sinapis Albae, Radix Euphorbiae Kansui, Herba Asari, and starch, at a ratio of 7:7:4:4:7. In parallel, subjects randomized to Group B were treated with placebo prepared by mixing the equal amount of starch and water. Notably, all herbs were purchased from Sichuan Traditional Chinese Medicine Co. LTD (Chengdu, China). Initially, the herbs were ground into powder (average size of 125 ± 5.8 µm) by WFM Ultra-Grinding Vibration Mill (Chengdu Saierte Machinery Co. LTD, Chengdu, China). Further, the mixture of herbs (10 g) was transformed into an ointment-like semi-solid dosage form using 2 mL of ginger juice. Then, the herbal semi-solid-like ointment was transferred inside a rubber ring on square tape. The rubber ring, with an approximate inner diameter of 2 cm and height of 1 mm, was placed in the middle of the square tape. The herbal ointment or placebo was plastered on 8 acupoints of BL, including bilateral BL13, BL17, BL20, and BL23. All subjects received the SAAT or sham treatment thrice per year on the hottest days in summer for 2 consecutive years (as shown in Table 1). When the subjects had potential adverse events or reported unbearable burning sensations, stinging, pain, and itching during treatment, they were allowed to peel off the ointment immediately and our acupuncturists would deal with the adverse events accordingly. Under normal conditions, the SAAT or sham treatments were performed for 4 hours each time.

**Table 1:** The dog day of the years 2018 to 2020.

Yr	Date (Dog day)		
2018	July 17	July 27	August 16
2019	July 12	July 22	August 11
2020	July 16	July 26	August 15

### 2.4. Sample collection and gut microbiota analysis

Fecal samples were collected twice before and after SAAT or sham treatments for participants, shipped on dry ice within half an hour after collection, and then frozen at −80°C freezer for analysis. Microbial DNA was extracted from human samples using the HiPure Soil DNA Kit B (Magen Biotech., Guangzhou, China) according to the manufacturer's protocol. The extracted DNA was quantified and the V3 and V4 hypervariable regions were amplified by polymerase chain reaction (PCR) system (ABI GeneAmp® 9700, Applied Biosystems, Waltham, MA) with the primers containing the forward sequence of “CCTACGGRRBGCASCAGKVRVGAAT” and the reverse sequence of “GGACTACNVGGGTWTCTAATCC.” The PCR reactions were conducted using the following program: 3 minutes of denaturation at 95°C, 27 cycles of 30 seconds at 95°C, 45 seconds for annealing at 55°C, and 45 seconds for elongation at 72°C, and final extension at 72°C for 10 minutes. PCR products were purified and quantified by the agarose gel electrophoresis with 1.5% agarose gel. Then the indexed adapters were added to the ends of the 16S rDNA amplicons to generate the indexed libraries ready for downstream next-generation sequencing sequencing on lllumina Miseq (Illumina, San Diego, CA). The concentrations of DNA libraries were validated by Qubit3.0 Fluorometer (Invitrogen, Waltham, MA, USA). After quantifying the library to 10 nM, the DNA libraries were multiplexed and loaded on an Illumina MiSeq instrument according to the manufacturer’s instructions (TruSeq™ DNA Sample Prep Kit, Illumina, San Diego, CA). Afterward, the sequencing was performed using the paired-end approach. Further, the image analysis and base calling were conducted by the MiSeq Control Software embedded in the MiSeq instrument. The positive and negative reads were joined together to filter the results contained in the sequence of N and the sequence length larger than the 200 bp sequence was retained. The retained sequences were qualitatively filtered and the chimeric sequences were deleted, and the sequence operational taxonomic unit (OTU) cluster analysis was then performed using VSEARCH (1.9.6) with the similarity set to 97%. The reference taxonomy was SILVA release 132. The Ribosomal Database Program Classifier Bayesian Algorithm of the OTU species taxonomy was then used to analyze representative sequences and under different species classification levels, statistical community compositions of each sample were performed. Microbiota alpha diversity was assessed by using the abundance-based coverage estimator and Chao1 indices of species richness and the Shannon and Simpson indices of diversity. Next, we assessed the β diversity of gut microbiota in diverse groups using principal component analysis (PCA), unweighted principal coordinate analysis (PCoA), and weighted distance matrices (nonmetric multidimensional scaling, NMDS). Finally, Linear discriminant analysis coupled with effect size (LEfSe) was performed with LEFSE software (LEfSe 1.0) to identify potential microbial biomarkers between groups.

### 2.5. Statistical analysis

Statistical analyses for clinical data were performed by SPSS software version 25 for Windows (SPSS, Chicago, IL). The categorical variables were analyzed using chi-squared test or Fisher exact test and expressed as frequency (percentage), while the continuous variables were expressed as mean ± standard deviation and compared with the Student *t* test or Mann–Whitney test as appropriate. The intestinal microorganisms were analyzed with the programming languages R (version 3.6.1). The alpha-diversity and relative abundance of the genera and species of gut microbiota were analyzed using Wilcoxon signed-rank/rank-sum test and expressed as median (interquartile range). For beta diversity analysis, the Bray–Curtis algorithm with PCOA, PCA, and NMDS was used to analyze the intergroup differences of the microbiota. An analysis of similarity was used for testing the significance of dissimilarity between the 2 groups by applying the read abundance. In addition, linear discriminant analysis of the effect size (LEfSe) and MetaStat were used to determine differences among the groups. The cladogram diagram showed the hierarchy of evolution between the group differences of microbial community structure and species. The difference of 0.05 was considered statistically significant when the *P* value < 0.05.

## 3. Results

A total of 72 subjects participated in the study and 60 (29 from Group A and 31 from Group B) participants completed the clinical trials. The remaining 12 patients dropped out for the following reasons: 2 participants from Group A withdrew due to adverse events including hyperpigmentation, allergic to herbs, or burning sensation in the skin, 5 subjects (1 from Group A and 4 from Group B) withdrew due to pregnancy, and 5 subjects (2 from Group A and 3 from Group B) withdrew before the end of the treatments due to personal reasons. In total, 130 stool samples were obtained from participants, of which 60 subjects provided 2 specimens before and after treatment, 10 subjects provided only pretreatment specimens, and the rest 2 subjects did not provide any samples.

### 3.1. Baseline clinical characteristics

Of the 72 participants enrolled in this analysis, there were 11 men and 23 women in group A (average age 43.00 ± 10.79 years) and 10 men and 28 women in group B (average age 37.24 ± 13.67 years). As shown in Table 2, there were no other significant differences in baseline characteristics between these 2 groups.

**Table 2:** The basic information baseline of the 2 groups of subjects was compared (mean ± SD).

Group	Gender		Average age (yr)	Height (cm)	Weight (kg)	Heart rate (beats/min)	Blood pressure (mm Hg)	
	Men	Women					Systolic	Diastolic
Group A(n = 34)	11	23	43.00 ± 10.79	162.06 ± 7.57	59.12 ± 9.23	74.88 ± 9.90	116.68 ± 10.88	75.12 ± 8.82
Group B(n = 38)	10	28	37.24 ± 13.67	159.42 ± 8.84	56.67 ± 8.91	73.97 ± 9.87	112.63 ± 11.42	73.13 ± 9.44
*P* value	.57		.05	.18	.26	.70	.13	.36

### 3.2. Sequencing analysis and OTU clustering

In total, 6994,402 sequences were obtained from the 130 samples through 16S rDNA high-throughput sequencing analysis. Furthermore, 510 bacterial OTUs were obtained, with a limited number at the 97% similarity cutoff level (as shown in Table 3). Raw reads were submitted to the National Center for Biotechnology Information Genbank, under project accession number PRJNA766254.

**Table 3:** The effective sequences and an average length of samples.

Index	Group	Before intervention			After intervention		
		N	Mean ± SD	*P* value	N	Mean ± SD	*P* value
Effective sequences	A	32	60042.72 ± 12772.94	.695	29	50403.83 ± 12991.60	.061
	B	38	58576.47 ± 17519.82		31	44690.90 ± 10055.65	
Average length (bp)	A	32	446.62 ± 5.17	.998	29	444.93 ± 2.99	.382
	B	38	446.61 ± 5.22		31	444.21 ± 3.34	

### 3.3. Alpha diversity analysis

**Figure 1.**
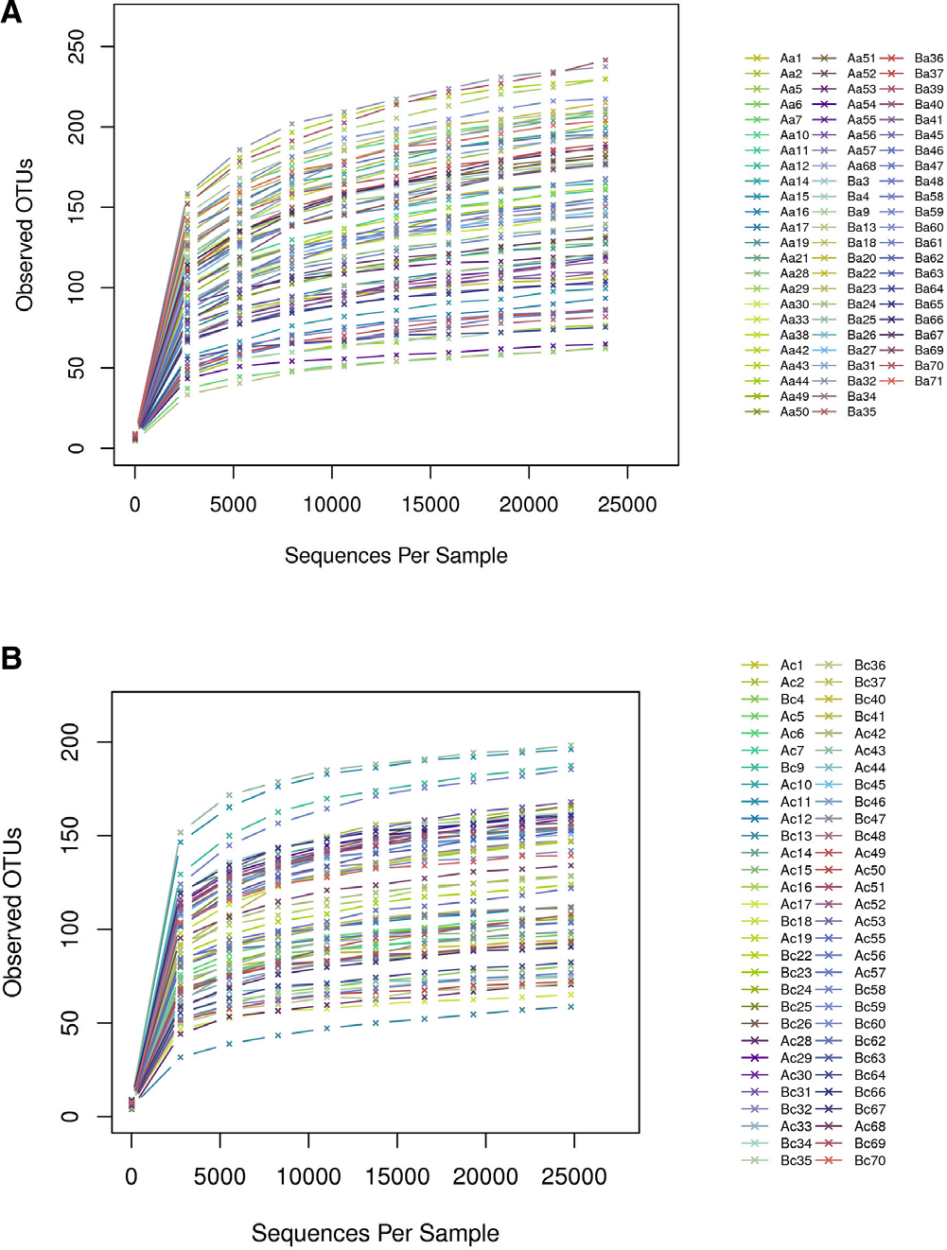
The graphical representation shows the rarefaction curve of each sample. The X-axis shows the effective sequences per sample. Y-axis shows the observed OTUs per sample. Aa12–Aa61 represent the samples’ names before intervention. Ab12–Ab61 indicate the samples’ names after the intervention. In the legend, Aa and Ac represent the total samples of Group A before and after the intervention, respectively. Ba and Bc represent the samples of Group B before and after the intervention. The legends of the later figures indicating the curve names are the same, respectively. OTU = operational taxonomic unit.

The values of Good coverage of all libraries were over 99.9%, indicating that the sample sequencing was relatively complete. Moreover, no significant differences were observed in the evaluated alpha diversity indexes (Shannon, Simpson, Chao1) between the groups. Rarefaction curves indicated that the sequencing depth was enough since the samples reached the plateau phase (Fig. 1). In addition, the number of sequenced reads of each sample was adequate to detect most species in the sample.

### 3.4. Beta diversity analysis

**Figure 2.**
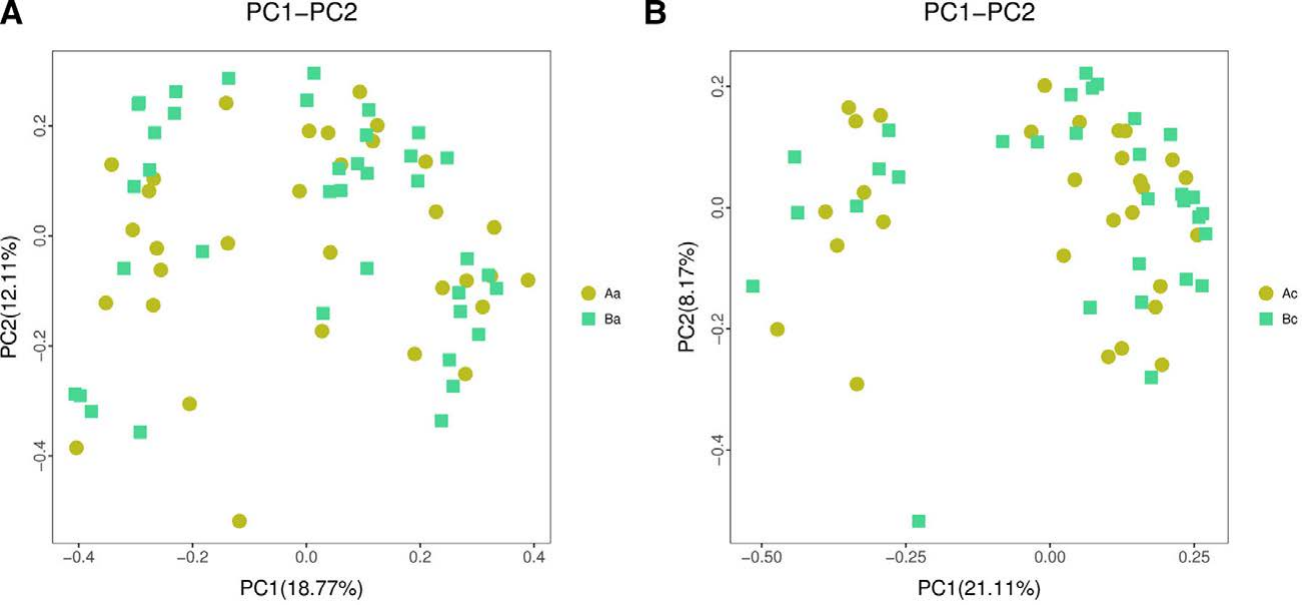
The plots indicate the PCoA analysis of 2 groups before and after the intervention. X-axis and Y-axis represent the first 2-component scores. PCoA = principal coordinates analysis.

**Figure 3.**
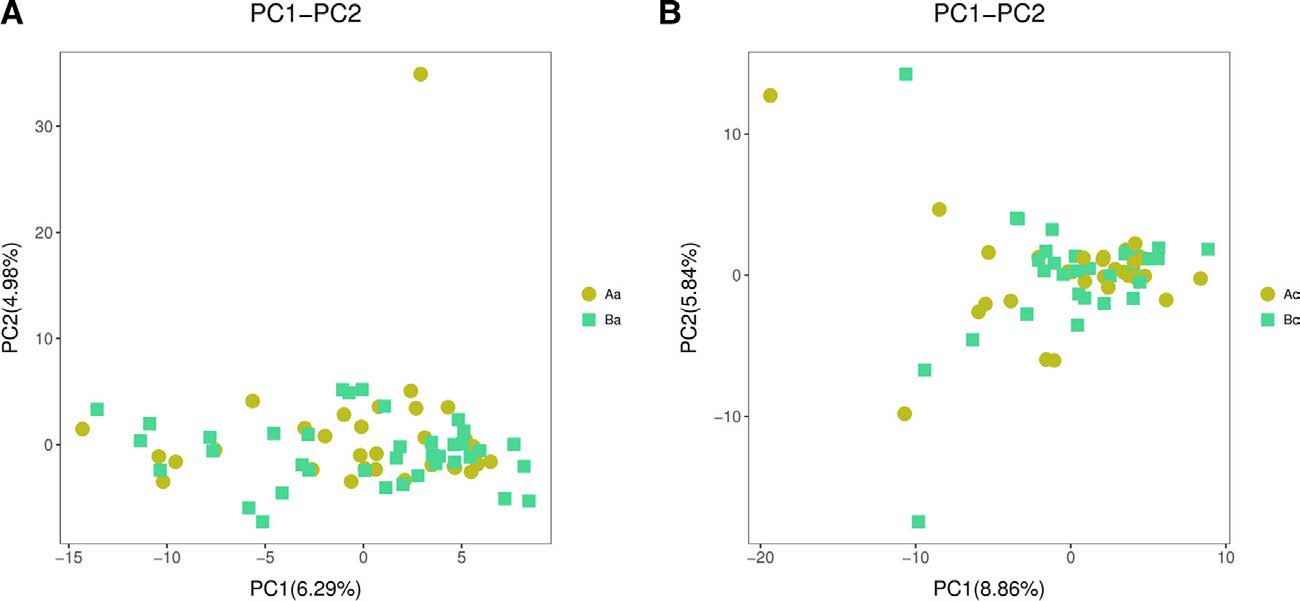
The plots present the PCA analysis of 2 groups before and after the intervention. X-axis and Y-axis represent the first 2-component scores. PCA = principal component analysis.

**Figure 4.**
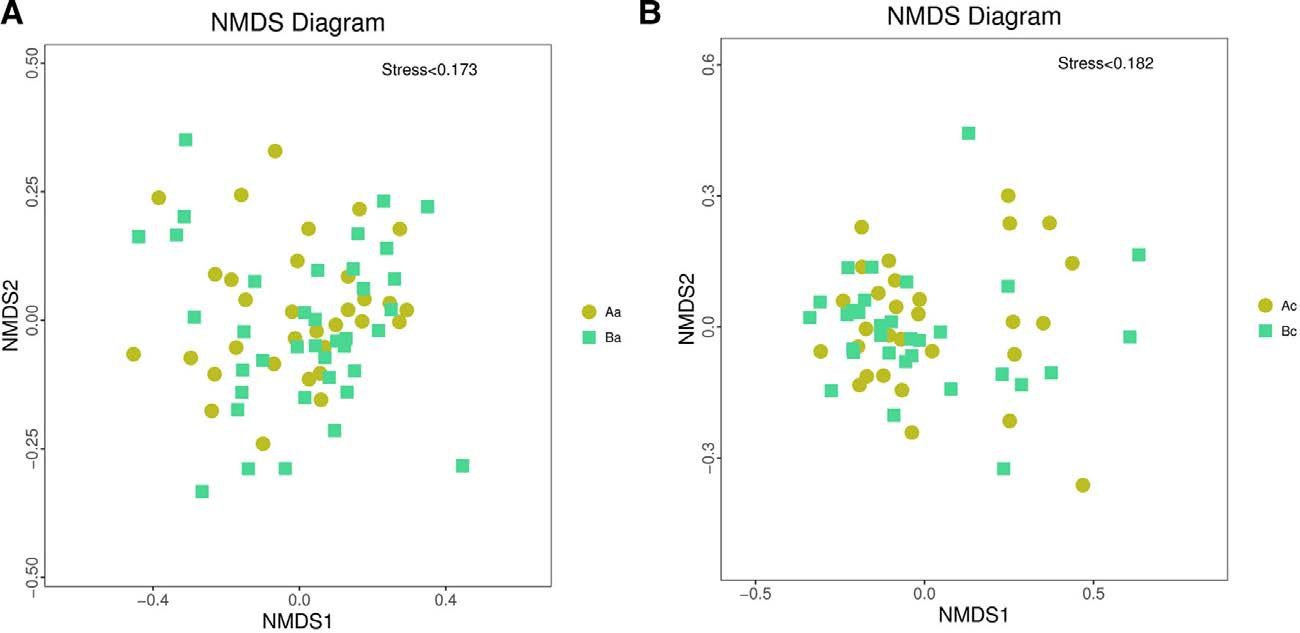
The plots indicate the NMDS analysis of 2 groups before and after the intervention. NMDS = non-metric multi-dimensional scaling.

Structural similarity was explored with the PCoA of the beta diversity analysis, which contained the first 2 principal coordinate axes. There was no appreciable change in the 2 groups before and after intervention (Fig. 2). Subsequently, the PCA analysis also revealed differences between the 2 groups based on the first 2 principal component scores. The data of the 2 groups also showed no segregation along axes regardless of before or after intervention (Fig. 3). The NMDS analysis (non-metric multidimensional scaling) depicted the intra-group dispersion of samples, indicating no significant difference between the 2 groups before or after intervention (Fig. 4).

### 3.5. Microbial structure characteristics

#### 3.5.1. LEfSe analysis.

**Figure 5.**
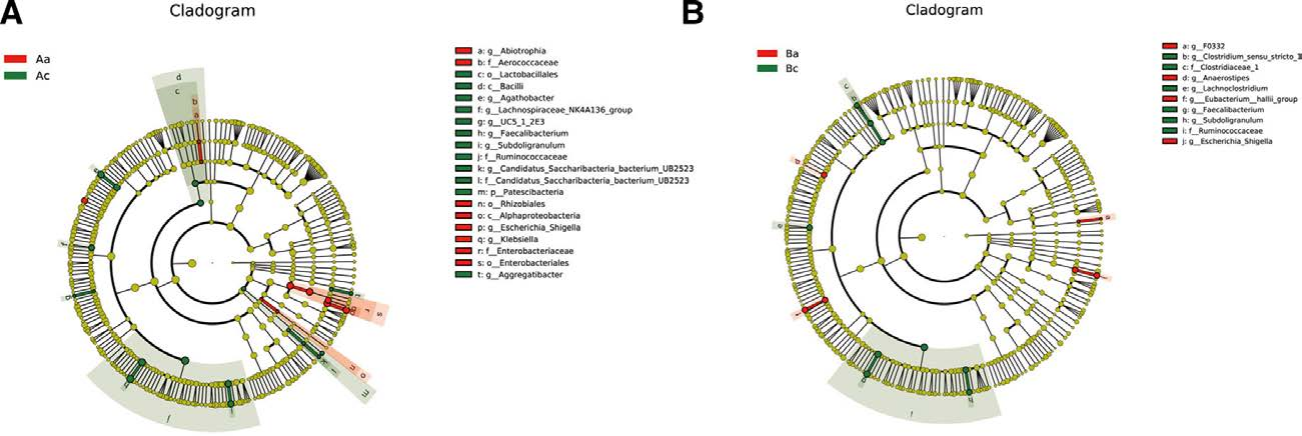
The graphical representations indicate the changes in gut microorganisms by SAAT and sham SAAT. In the phylogenetic tree diagrams of species clustering, green nodes represent microorganisms that play essential roles in the green group. The red nodes represent microorganisms that play significant roles in the red group. Contrarily, the yellow nodes represent microorganisms that do not play essential roles in both groups. SAAT = summer acupoint application treatment.

In general, the LEfSe analysis provides a phylogenetic tree diagram of clustered species, presenting the changes in the important microorganisms between the different treatment groups. Remarkably, 20 microbiota species were found to have a changed relative abundance before and after SAAT therapy in Group A (Aa vs Ac, Fig. 5A). Furthermore, we also observed an alteration of 10 microbiota species in Group B after 2 years of sham treatment (Ba vs Bc, Fig. 5B). Accordingly, we could speculate that alteration of intestinal microflora was more common in SAAT treatment group as compared to sham treatment group.

#### 3.5.2. Analysis of the structure of bacterial phyla.

**Figure 6.**
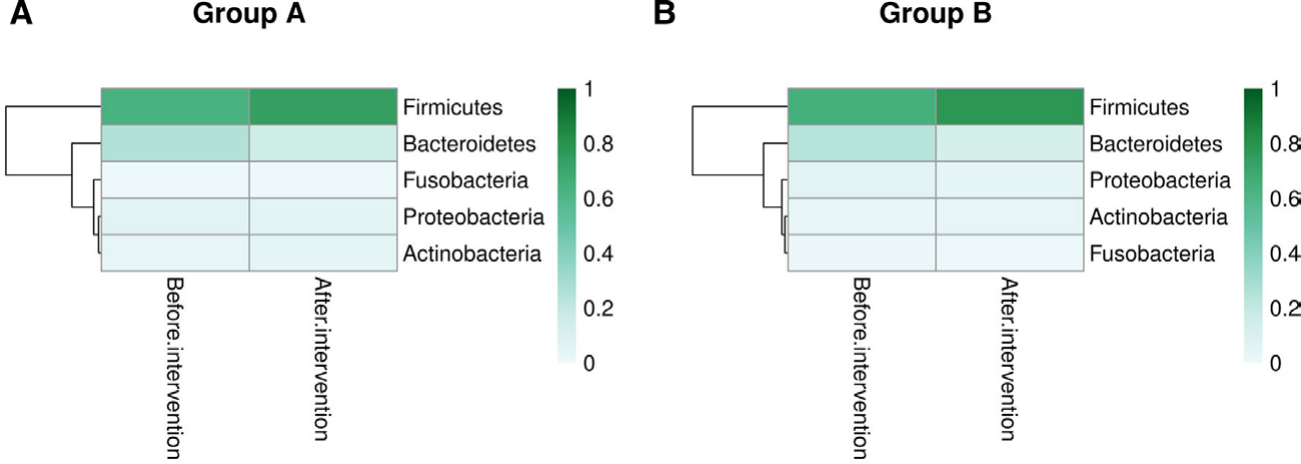
The heatmap shows the 5 main bacterial phyla of 2 groups before and after the intervention.

At the phylum level, the baseline relative abundance of *Firmicutes, Bacteroidetes, Proteobacteria, Actinobacteria*, and *Fusobacteria* was identified in fecal samples collected from each group (Fig. 6). The statistical difference for the abundance of gut microbiota at the phylum level was insignificant between the 2 groups before treatment. Among various bacterial phyla, *Firmicutes* was the most abundant phylum before treatment in both groups, which accounted for 62.68% in Group A and 65.28% in Group B. After 2 years of treatment, the relative abundance of *Firmicutes* increased to 73.99% in Group A (*P* = .042) and 74.82% in Group B (*P* = .016). Furthermore, at the completion of treatment, the relative abundance of *Fusobacteria* in Group A was reduced from 0.0078 ± 0.0006 to 0.0005 ± 0.0000 (*P* < .001), while the relative abundance of *Bacteroidetes* was decreased from 0.2395 ± 0.0750 to 0.1300 ± 0.0182 (*P* = .037) in Group B. Whereas there were no significant differences in the relative abundances of other bacterial phyla before and after treatment (*P* > .05).

#### 3.5.3. Analysis of the structure of bacterial genera.

**Figure 7.**
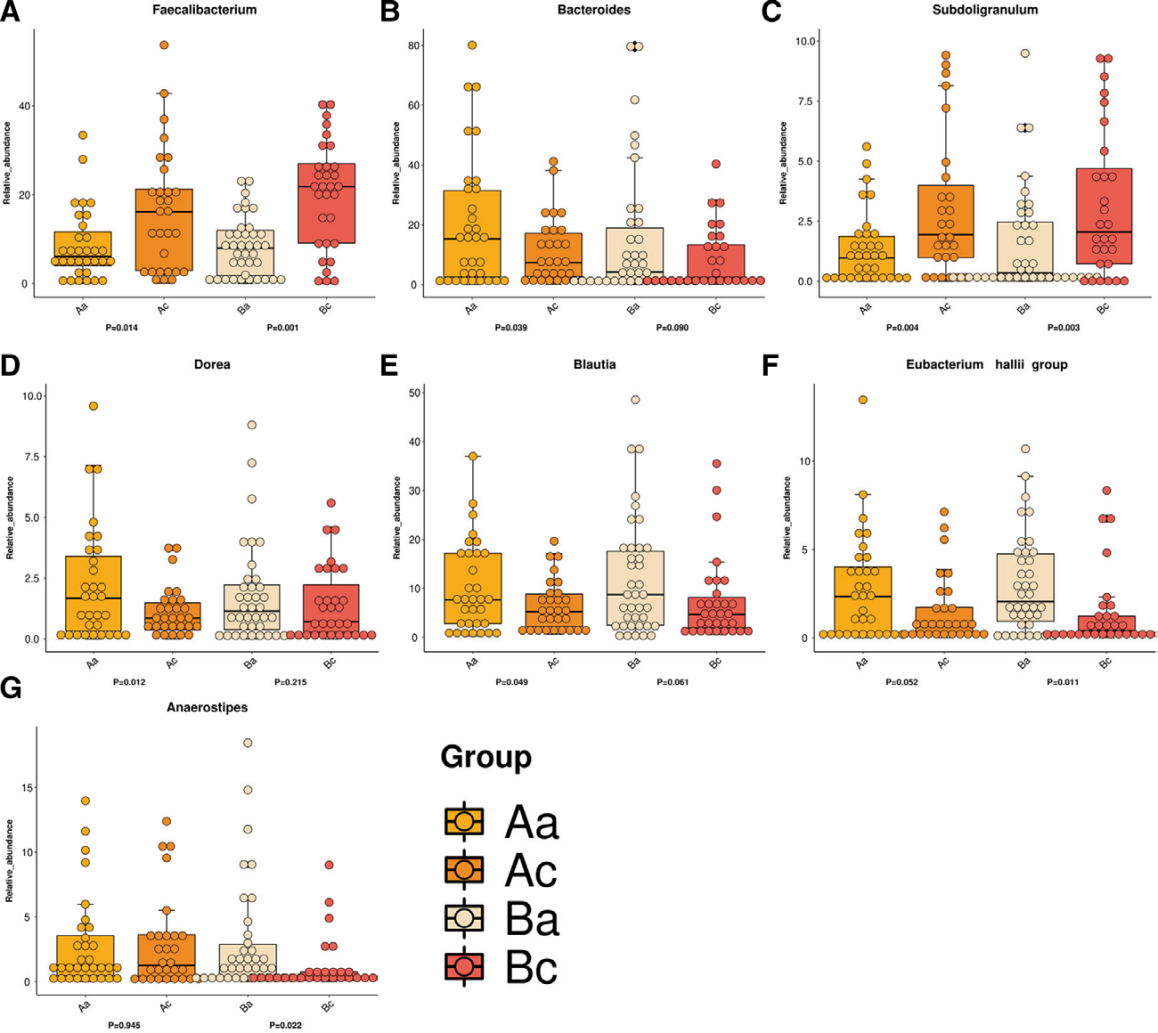
The graphs indicate the difference between the bacterial genera in the 2 groups before and after intervention (relative abundance values >0.01).

At the genus level, As depicted in Figure 7, the relative abundances of 2 genera including *Faecalibacterium* and *Subdoligranulum* were significantly increased from 0.085 ± 0.0142 to 0.1622 ± 0.0256 (*P* = .014) and from 0.085 ± 0.0142 to 0.1622 ± 0.0256 (*P* = .014) in Group A after treatment completion, respectively. Conversely, the relative abundances of 3 genera including *Dorea, Bacteroides*, and *Blautia* significantly reduced from 0.0245 ± 0.0048 to 0.0115 ± 0.0019 (*P* = .012), 0.2004 ± 0.0391 to 0.1102 ± 0.0205 (*P* = .039) and 0.1021 ± 0.0163 to 0.0653 ± 0.0096 (*P* = .049) in Group A, respectively. As for Group B after completion of treatment, the relative abundances of genera *Faecalibacterium* and *Subdoligranulum* were substantially increased from 0.0828 ± 0.011 to 0.2001 ± 0.0217 (*P* = .001) and from 0.0152 ± 0.0036 to 0.0451 ± 0.0102 (*P* = .003), respectively. Contrarily, the relative abundances of the *Eubacterium hallii* and *Anaerostipes* genera were significantly decreased from 0.0297 ± 0.0045 to 0.0139 ± 0.0039 (*P* = .011) and from 0.0293 ± 0.007 to 0.0111 ± 0.0037 (*P* = .022), respectively.

## 4. Discussion

In this randomized, placebo-controlled study, we evaluated the potential effect of SAAT on fecal microbiota composition in healthy adults by 16S rDNA sequencing. Generally, our present investigation first revealed the relative abundance of the *Fusobacteria* phylum declined significantly after 2 years of treatment with SAAT. To our knowledge, the relative increase of bacterial taxa in the Phylum *Fusobacteria* is associated with periodontal disease^[33]^ and inflammatory bowel disease^[34]^ in humans, which implies that SAAT might change the composition of the gut microbiota to potentially ameliorate colitis-associated diseases.^[35]^ Concomitantly, we observed the proportion of *Bacteroidetes* was decreased in Group B, resulting in an increasing trend in the *Firmicutes*-*Bacteroidetes* ratio (F/B ratio). The F/B ratio has been widely used to indicate microbial dysbiosis^[36]^ and the ratio was lower in Group A as compared to Group B at the completion of treatment (4.59 vs 6.03). The increased F/B ratio, caused by an expansion of *Firmicutes* and/or a contraction of *Bacteroidetes*, has been considered a promising modifiable risk factor for obesity and type 2 diabetes,^[37]^ which was well validated in rodent and human samples.^[38]^ Our results with regard to phylum-level changes indicated SAAT might serve as a precautionary measure for metabolic diseases and the specific mechanism needs further exploration.

After the 2-year SAAT treatment, the relative abundance of *Blautia, Bacteroides*, and *Dorea* decreased, while the relative abundance of *Faecalibacterium* and *Subdoligranulum* increased at the genus level, and the proportion of the *Eubacterium hallii* and *Anaerostipes* remained relatively constant. *Blautia* and *Faecalibacterium* are short-chain fatty acid-producing bacteria (acetate and butyrate, respectively).^[39]^ Data from animal studies show that increasing acetate production via regulation of gut microbiota including *Blautia* and *Dorea* could promote ghrelin secretion, hyperphagia, obesity, and its related sequelae.^[40]^ Alternatively, this class of bacteria has been strongly linked to the pathogenesis of acute graft-versus-host disease.^[41]^ Remarkably, less abundant butyrate-producing bacteria including *Faecalibacterium* along with reduced butyrate formation were observed in inflammatory bowel disease and type 2 diabetes individuals.^[42]^ Consistent with previous studies that demonstrated positive effects of acupuncture on gut microbiota,^[28]^ we hypothesized that SAAT treatment could increase the abundance of beneficial bacterium *Faecalibacterium* and reduce harmful bacteria, modulating intestinal-immune system and ameliorating inflammation, thereby achieving the purpose of preventing chronic diseases in healthy population. More recently, a study by Bao et. al suggested that acupuncture may increase the abundance of short-chain fatty acids producing bacteria and anti-inflammatory bacteria including *Faecalibacterium*, thereby enhancing intestinal barrier function, and inhibiting Th1/Th17 cells related proinflammatory cytokines, which provided a safe, effective therapeutic manner for patients with mild to moderate Crohn disease.^[28]^ Research by Wang et al found electroacupuncture could decrease the body weight, waist circumference, and visceral adipose tissues of obese rats by regulating *Firmicutes*/*Bacteroidetes* ratio, thereby improving insulin sensitivity, glucose homeostasis, and lipid metabolism.^[43]^ Xu et al explored the impact of acupuncture on intestinal bacteria in osteosarcoma tumor-burdened mice, revealing acupuncture treatment delayed the decrease of *Bacteroidetes* and the increase of *Firmicutes*, and the tumor growth in mice-burdened osteosarcoma.^[44]^ The results of Yu et al also suggested that warm acupuncture could regulate a variety of microbial genera and metabolites related to insomnia, including *Blautia*, reversing the butyrate-mediated upregulation of the cAMP signaling pathway and GAT-1 expression.^[45]^ Collectively, this study is among the very few suggesting that SAAT could modulate the intestinal microbiota and serve as a measure to prevent inflammation and metabolism-associated diseases, whereas the mechanisms underlying SAAT regulating the intestinal microflora remain to be elucidated.

According to the TCM theory, the health of a person depends on the dynamic balance between the physiological state and the surrounding environment. Recently, the gut microbiota has also been identified as an important link for balance.^[18]^ In disease states, however, this holistic balance was disrupted, and SAAT combining points and meridians with drugs were used for triggering a comprehensive and systemic adjustment to balance the disruption.^[43]^ Chan et al performed a meta-analysis and found that *san fu tian* (a form of SAAT) therapy has positive effects on adult asthma and this technology is relatively safe because of its noninvasive nature.^[15]^ The results of Shi et al indicated SAAT with Shenhuang plaster reshaped the composition of the microbiota, especially butyrate-producing gut bacterium, and promotes intestinal peristalsis, and the combination of paclitaxel with Shenhuang SAAT exerted potent immunostimulatory effects.^[23]^ At present, there have been several studies applying SAAT or other forms of acupuncture to the remission of coronavirus disease 2019-related symptoms, including headache,^[46]^ diarrhea,^[47]^ and fatigue.^[48]^ Whereas, few studies have focused on the mechanisms underlying the prophylactic effect of SAAT.^[49]^ In this study, we addressed the potential role of SAAT in maintaining intestinal microbiota homeostasis and the prevention of inflammatory and metabolic disease. It is necessary to carry out long-term follow-up studies in the future to explore the effect of SAAT on the prevention and rehabilitation of chronic conditions, including the sequelae of COVID-19.^[50]^

There are several limitations that should be acknowledged in this study. First, we had a limited number of participants, which might lead to subject selection bias. Second, the extended follow-up has not been assessed and the long-term effect of acupuncture on these indices could not be determined. Third, we only included healthy individuals without enrolling in disease groups, which limits our ability to infer the modulation effect of SAAT on gut microbiome disorders. Finally, we did not perform the metabolomics study of feces and the combination of fecal metabolomics and 16S rDNA gene sequencing would be the future direction of our research. Nonetheless, the present trial is the first randomized, controlled study to evaluate the impact of SAAT on the abundance and biological structure of gut microbiota in healthy Asian adults, which needs further validation in larger cohorts.

## 5. Conclusion

In summary, this study has illustrated that the SAAT substantially influenced the bacterial community structure in the gut microbiota of healthy adults, which might serve as potential therapeutic targets for related diseases, and provided a foundation for future studies aimed at elucidating the microbial mechanisms underlying SAAT for the treatment of inflammatory and metabolic disease. However, our findings should be interpreted with some caution, and the practitioner must reconsider the indications and specifications of SAAT while applying it to healthy people despite its noninvasive nature. In the long term, our findings are important for understanding the positive effect of SAAT on the host intestinal microbiota, and further research should combine microbiome and metabolome data to further assess the molecular effects of SAAT on the prevention of inflammatory and metabolic diseases.

## Acknowledgments

We thank the Science and Technology Department of Sichuan Province, Sichuan Provincial Administration of Traditional Chinese Medicine for the funding.

## Author contributions

**Conceptualization:** Jie Zhou, Bangmin Zhou, Xianbo Wu.

**Data curation:** Bangmin Zhou, Xiaoyue Kou, Shijian Jia.

**Investigation:** Xiaoyue Kou, Tao Jian.

**Methodology:** Xiaoyue Kou, Tao Jian, Xiaoying Xie.

**Project administration:** Tao Jian, Xiaoying Xie.

**Software:** Jie Zhou, Bangmin Zhou.

**Supervision:** Limei Chen, Xinghua Lei, Xianbo Wu.

**Validation:** Limei Chen, Xinghua Lei.

**Visualization:** Jie Zhou, Bangmin Zhou.

**Writing – original draft:** Jie Zhou, Bangmin Zhou.

**Writing – review & editing:** Xianbo Wu.
